# Effects of Nabilone on Sleep Outcomes in Patients with Parkinson's Disease: A Post‐hoc Analysis of NMS‐Nab Study

**DOI:** 10.1002/mdc3.13471

**Published:** 2022-05-31

**Authors:** Marina Peball, Klaus Seppi, Florian Krismer, Hans‐Günther Knaus, Sabine Spielberger, Beatrice Heim, Philipp Ellmerer, Mario Werkmann, Werner Poewe, Atbin Djamshidian

**Affiliations:** ^1^ Department of Neurology Medical University of Innsbruck Innsbruck Austria; ^2^ Department for Medical Genetics, Molecular, and Clinical Pharmacology Medical University of Innsbruck Innsbruck Austria

**Keywords:** cannabinoids, nabilone, sleep problems, Parkinson's disease, non‐motor symptoms

## Abstract

**Background:**

The synthetic tetrahydrocannabinol analogue nabilone improved overall non‐motor symptom (NMS) burden in Parkinson's disease (PD) patients in comparison to placebo.

**Objectives:**

To characterize the effects of nabilone on different sleep outcomes in PD patients.

**Methods:**

We performed a post‐hoc analysis of the controlled, double‐blind, enriched enrollment randomized withdrawal NMS‐Nab study to assess the effects of nabilone on sleep outcomes in study participants who reported clinically‐relevant sleep problems (MDS‐UPDRS‐1.7 ≥ 2 points).

**Results:**

After open‐label nabilone administration, 77.4% reported no relevant sleep problem. In the withdrawal phase of the trial, the MDS‐UPDRS‐1.7. and the NMS‐Scale Domain 2 (i.e., Sleep/Fatigue) significantly worsened only in PD patients in the placebo group, which was mostly driven by a significant worsening of insomnia (question 5 of the NMS‐Scale Domain 2).

**Conclusions:**

This post‐hoc analysis of the NMS‐Nab trial suggests that nabilone has beneficial effects on sleep outcomes in PD patients experiencing sleep problems at baseline.

The original trial was registered with ClinicalTrials.gov (NCT03769896, https://clinicaltrials.gov/ct2/show/NCT03769896) and EudraCT (2017–000192‐86).

Sleep problems are among the most common non‐motor symptoms (NMS) in Parkinson's disease (PD) and adversely affect the patient's quality of life and daily functioning.^1^, ^2^ We have recently studied the efficacy and safety of the synthetic Delta‐9‐tetrahydrocannabinol (THC) analogue nabilone in PD patients with troublesome NMS in a placebo‐controlled, double‐blind (DB), parallel‐group, enriched‐enrollment‐randomized‐withdrawal (EERW) trial (NMS‐Nab trial).^3^ We found that patients who switched to placebo experienced significant worsening of NMS compared to those remaining on nabilone. Positive treatment effects of nabilone were also reflected in the patient's Clinical‐Global‐Impression of Improvement Scale, mainly driven by reduced anxiety and sleep problems.^3^

Based on these results, this post‐hoc analysis was performed to explore the effects of nabilone on clinically‐relevant sleep problems^4^, ^5^ in PD (termed as “symptomatic”) Fig. 1.

**FIG. 1:**
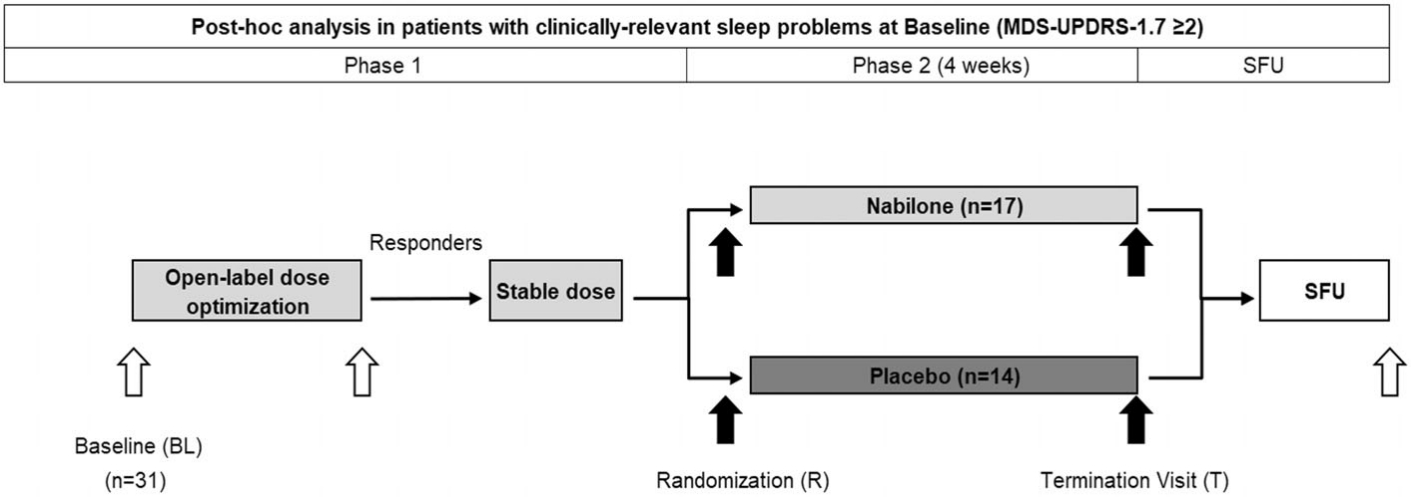
Schedule of trial activities. All patients received nabilone during phase 1 of the trial. Abbreviations: BL, Baseline; R Randomization; T, Termination Visit; SFU, Safety Follow‐Up.

## Methods

The study was approved by the local ethics committee and the Austrian national regulatory authorities (ClinicalTrials.gov NCT03769896, registration date: December 10, 2018) and EudraCT (2017–000192‐86, registration start date: September 15, 2017). All individuals gave written informed consent before participation. All procedures were performed in accordance with the Helsinki declaration. The study design and results were reported elsewhere.^3^, ^6^ Shortly, PD patients with stable motor disease suffering from disturbing NMS defined as a total score of ≥4 points on the MDS‐UPDRS‐1 and a score of ≥2 points on either MDS‐UPDRS‐1.4 (anxious mood) or 1.9 (pain) were included. Presence of sleep problems was not an inclusion criterion for the NMS‐Nab trial.^3^

### Post‐hoc Analysis

This post‐hoc analysis included 31 of the 38 randomized PD patients who were symptomatic with clinically‐relevant sleep problems at baseline, defined as a score of ≥2 points in item 1.7 of the MDS‐UPDRS. The MDS‐UPDRS‐1.7 captures questionnaire‐based patient information and a score of 2 corresponds to “mild” sleep problems which usually cause some difficulties getting a full night of sleep, considered therefore clinically‐relevant in recent studies.^4^, ^5^

The sleep‐related outcome measures used for this post‐hoc analyses included MDS‐UPDRS items 1.7 (sleep problems, 0–4 points) and 1.8 (daytime sleepiness, 0–4 points), the NMSS Domain 2 (NMSS‐D2, Sleep/Fatigue, 0–48 points), the single questions (Q) of the NMSS‐D2 (each 0–12 points), and the Epworth Sleepiness Scale (ESS, 0–24 points). Higher score values indicate worse outcome in all scores. Baseline refers to study inclusion (i.e., screening), randomization to the start of the DB phase, and termination visit to the end of it.

### Statistical Analyses

A descriptive analysis of demographic and clinical data at baseline was performed in all patients randomized for the DB EERW part of the NMS‐Nab trial.^6^ We used the Wilcoxon matched‐pairs test for within‐group comparison during the open‐label (OL) phase and during the DB phase, the Wilcoxon matched‐pairs test for within‐group comparison (correction for multiple comparisons with a factor of two) and a Mann–Whitney U test for between‐group comparisons. Statistical significance was set at a two‐sided 5% α‐level. Effect sizes for the different endpoints were calculated according to Cohen's D with Hedges’ g correction and Common Language Effect Size (CLES). Cohen's D of 0.2–0.5, 0.5–0.8, and >0.8 was considered a “small,” “medium,” and “large” effect size.^7^ In between‐group comparisons, CLES represents the probability that a randomly sampled patient from one group will have a higher score than a random patient from the other group.^8^ As a probability‐based metric, CLES ranges from 0 to 1. We have interpreted CLES values of 0.56–0.64, 0.64–0.71, and >0.71 as “small,” “medium,” and “large” effects.^9^ SPSS 25.0 for windows (SPSS Inc., IBM Corporation and other(s) 1989, 2017, Chicago, IL, USA) was used to analyze data. Effect sizes were calculated as described elsewhere.^7^

## Results

A total of 14/31 patients were randomized to placebo and 17 to nabilone in the DB phase. For clinical characteristics see Table 1.

**TABLE 1:** Characteristics of the study population at baseline

	Full data set (n = 38)	PD patients with clinically‐relevant sleep problems (MDS‐UPDRS‐1.7 ≥ 2, n = 31)			*P*‐value*
	Baseline	Baseline	Placebo Group (n = 14)	Nabilone Group (n = 17)	
Age (in years)	64.66 ± 7.92, 66.17	64.34 ± 8.14, 65.92	63.51 ± 8.17, 64.54	65.02 ± 8.30, 66.83	0.617
Females	14 (36.8%)	12 (38.7%)	3 (21.4%)	9 (52.9%)	0.073
Disease duration	7.61 ± 5.24, 6.00	7.50 ± 5.17, 6.00	6.87 ± 4.54, 5.50	8.01 ± 5.72, 7.25	0.550
Daily nabilone dose (mg)^a^	0.86 ± 0.40, 0.75 (0.25–1.75)	0.90 ± 0.42, 1.00 (0.25–1.75)	0.86 ± 0.44, 0.75 (0.25–1.50)	0.94 ± 0.42, 1.00 (0.25–1.75)	0.589
MDS‐UPDRS‐1	12.90 ± 5.14, 12.00	13.84 ± 5.03, 13.00	13.79 ± 5.81, 12.00	13.88 ± 4.49, 15.00	0.959
MDS‐UPDRS‐1.7	2.50 ± 1.11, 2.00	2.87 ± 0.85, 3.00	2.79 ± 0.80, 3.00	2.94 ± 0.90, 3.00	0.619
MDS‐UPDRS‐1.8	1.08 ± 0.88, 1.00	1.16 ± 0.90, 1.00	1.43 ± 0.76, 1.00	0.94 ± 0.97, 1.00	0.135
NMSS Domain 2	13.29 ± 8.29, 11.50	15.32 ± 7.72, 14.00	15.43 ± 7.87, 15.00	15.24 ± 7.83, 14.00	0.946
ESS	8.00 ± 3.95, 8.00	8.23 ± 4.09, 8.00	7.86 ± 4.04, 7.50	8.53 ± 4.23, 8.00	0.656
0–5 points	9 (23.7%)	7 (22.6%)	3 (21.4%)	4 (23.5%)	0.922
6–10 points	21 (55.3%)	17 (54.8%)	8 (57.1%)	9 (52.9%)	
11–12 points	3 (7.9%)	2 (6.5%)	1 (7.1%)	1 (5.9%)	
13–15 points	4 (10.5%)	4 (12.9%)	2 (14.3%)	2 (11.8%)	
16–24 points	1 (2.6%)	1 (3.2%)	0	1 (5.9%)	

Data are presented as mean ± standard deviation, median for continuous variables and number (percent) for categorical variables. Abbreviations: MDS‐UPDRS, Movement Disorder Society – Unified Parkinson's Disease Rating Scale; NMSS, Non‐Motor Symptoms Scale; ESS, Epworth Sleepiness Scale; MDS‐UPDRS‐1.7: Nighttime sleep problems, 1.8: Daytime sleepiness; NMSS Domain 2: Sleep/Fatigue.

^a^ Daily nabilone dose at the randomization visit in milligrams, mean ± standard deviation; median (minimum – maximum).

*P‐value represents the difference between the 31 patients of the placebo and nabilone groups. T‐test for continuous variables (all normally distributed), Qui‐square test for categorical variables. Significance level was set at p ≤ 0.05.

MDS‐UPDRS‐1.7 question: Sleep problems:

Over the past week, have you had trouble going to sleep at night or staying asleep through the night? Consider how rested you felt after waking up in the morning.

0: Normal: No problems. 1: Slight: Sleep problems are present but usually do not cause trouble getting a full night of sleep. 2: Mild: Sleep problems usually cause some difficulties getting a full night of sleep. 3: Moderate: Sleep problems cause a lot of difficulties getting a full night of sleep, but I still usually sleep for more than half the night. 4: Severe: I usually do not sleep for most of the night.

MDS‐UPDRS‐1.8 question: Daytime sleepiness:

Over the past week, have you had trouble staying awake during the daytime?.

0: Normal: No daytime sleepiness. 1: Slight: Daytime sleepiness occurs, but I can resist and I stay awake. 2: Mild: Sometimes I fall asleep when alone and relaxing. For example, while reading or watching TV. 3: Moderate: I sometimes fall asleep when I should not. For example, while eating or talking with other people.

4: Severe: I often fall asleep when I should not. For example, while eating or talking with other people.

ESS: How likely are you to doze off or fall asleep in the following situations, in contrast to feeling just tired? 0 = would never doze 1 = slight chance of dozing 2 = moderate chance of dozing 3 = high chance of dozing. Situations: Sitting and reading, Watching TV, Sitting, inactive in a public place (e.g. a theater or a meeting), As a passenger in a car for an hour without a break, Lying down to rest in the afternoon when circumstances permit, Sitting and talking to someone, Sitting quietly after a lunch without alcohol, In a car, while stopped for a few minutes in the traffic.

Both the MDS‐UPDRS‐1.7 and the NMSS‐D2 improved significantly during OL treatment with nabilone (*P* < 0.001, Table 2). OL administration of nabilone resulted in an amelioration of at least one point in 30 patients (96.8%) and of at least two points in 22 patients (71.0%) in the MDS‐UPDRS‐1.7. Consequently, 24 patients (77.4%) reported no relevant sleep problem (i.e., a score of 0 or 1 on the MDS‐UPDRS‐1.7) and 13 patients (41.9%) no sleep problems at all (i.e., a score of 0) at randomization. Scores of the NMSS‐D2 questions for difficulty falling or staying asleep (Q5, *P* < 0.001) and restless legs (Q6, *P* = 0.043) also decreased with OL nabilone (Table 2).

**TABLE 2:** Changes of MDS‐UPDRS‐1.7 (“sleep problems”), NMSS domain 2 (“sleep/fatigue”) and other outcome measures in PD patients with clinically‐relevant sleep problems during the trial

Open‐label Phase: Changes of MDS‐UPDRS‐1.7 (“sleep problems”) and NMSS Domain 2 (“sleep/fatigue”)						
		Baseline	Change from BL to R		*P*‐value^a^	
**MDS‐UPDRS‐1.7**		2.87 ± 0.85, 3.00	−1.97 (−2.30; −1.63)		<0.001	
**NMSS Domain 2**		15.32 ± 7.72, 14.00	−5.77 (−8.24; −3.31)		<0.001	
**MDS‐UPDRS‐1.7:** n (%) improved by ≥1 point and ≥2 points		30 (96.8%) and 22 (71.0%)				
n (%) with no clinically‐relevant sleep problems (i.e., **MDS‐UPDRS‐1.7** ≤ 1) at R		24 (77.4%)				
n (%) with no sleep problems (i.e., **MDS‐UPDRS‐1.7** = 0) at R		13 (41.9%)				

Open‐label Phase: Changes of other outcome measures						
		Baseline	Change from BL to R		*P*‐value^a^	
**NMSS Q3** (Daytime sleepiness)		1.39 ± 2.03, 0.00	−0.07 (−0.79; 0.66)		0.671	
**NMSS Q4** (Fatigue)		3.07 ± 3.13, 3.00	−0.52 (−1.40; 0.37)		0.263	
**NMSS Q5** (Insomnia)		7.84 ± 3.75, 8.00	−4.39 (−2.94; −5.84)		<0.001	
**NMSS Q6** (Restless legs)		3.03 ± 3.94; 1.00	0.81 (−0.02; −1.60)		0.043	
**MDS‐UPDRS‐1.8**		1.16 ± 0.90, 1.00	−0.16 (−0.45; 0.12)		0.251	
**ESS**		8.23 ± 4.09, 8.00	0.45 (−0.44; 1.34)		0.383	

Double‐blind Phase: Changes of MDS‐UPDRS‐1.7 (“sleep problems”) and NMSS Domain 2 (“sleep/fatigue”)						
		Randomization	Change from R to T (within‐groups)	*P*‐value^a^^,*^; Effect size **	Difference (between‐groups)	*P*‐value^b^; Effect size ***
**MDS‐UPDRS‐1.7**	P	0.79 ± 1.12, 1.00	2.00 (1.32; 2.68)	0.004; 1.70	1.88 (1.04; 2.73)	<0.001; 1.65/ 0.88
	N	1.00 ± 0.94, 1.00	0.12 (−0.45; 0.69)	1.000; 0.11		
**NMSS Domain 2**	P	7.57 ± 7.06, 6.00	9.57 (3.24; 15.90)	0.004; .87	8.57 (2.24; 14.91)	0.011; 1.00/ 0.76
	N	11.18 ± 8.43, 8.00	1.00 (−2.08; 4.08)	0.800; 0.17		
**MDS‐UPDRS‐1.7:** n (%) of patients that worsened by ≥1 point	P	12 (85.7%)			0.002 ****	
	N	5 (29.4%)				
**MDS‐UPDRS‐1.7:** n (%) of patients that worsened by ≥2 points	P	11 (78.6%)			<0.001 ****	
	N	1 (5.9%)				
**MDS‐UPDRS‐1.7 ≥ 2** at T in patients with no clinically‐relevant sleep problems (i.e., MDS‐UPDRS‐1.7 ≤ 1) at R (n = 24)	P	11 (91.7%)			<0.001 ****	
	N	2 (16.7%)				

Double‐blind Phase: Changes of other outcome measures						
		Randomization	Change from R to T (within‐groups)	*P*‐value^a^^,*^	Difference (between‐groups)	*P*‐value^b^
**NMSS Q3** (Daytime sleepiness)	P	1.21 ± 1.81, 0.00	0.71 (−0.08; 1.51)	0.078	0.19 (−0.67; 1.04)	0.773
	N	1.41 ± 2.65, 0.00	0.53 (0.04; 1.01)	0.082		
**NMSS Q4** (Fatigue)	P	2.14 ± 2.51, 1.00	1.64 (−0.26; 3.55)	0.186	0.70 (−1.48; 2.88)	0.595
	N	2.88 ± 3.06, 2.00	0.94 (−0.41; 2.30)	0.346		
**NMSS Q5** (Insomnia)	P	3.36 ± 3.59, 2.00	5.36 (2.89; 7.82)	0.006	5.00 (1.92; 8.09)	0.004
	N	3.53 ± 3.52, 3.00	0.35 (−1.75; 2.46)	1.000		
**NMSS Q6** (Restless legs)	P	0.86 ± 1.51, 0.00	1.86 (−0.71; 4.43)	0.442	2.68 (−0.12; 5.49)	0.265
	N	3.35 ± 4.54; 1.00	−0.82 (−2.15; 0.50)	0.394		
**MDS‐UPDRS 1.8**	P	1.07 ± 0.73, 1.00	0.21 (−0.12; 0.55)	0.360	−0.08 (−0.51; 0.35)	0.694
	N	0.94 ± 0.83, 1.00	0.29 (−0.01; 0.60)	0.118		
**ESS**	P	8.57 ± 4.59, 7.50	−0.64 (−1.85; 0.57)	0.536	−0.06 (−1.75; 1.64)	0.764
	N	8.76 ± 3.95, 8.00	−0.59 (−1.85; 0.67)	0.728		

Abbreviations: BL, baseline; R, randomization; T, termination visit; P, Placebo; N, Nabilone; MDS‐UPDRS, Movement Disorder Society‐ Unified Parkinson's Disease Rating Scale; NMSS, Non‐Motor Symptoms Scale; 1.7, MDS‐UPDRS‐1 item 1.7 (Sleep problems); D2, NMSS Domain 2 (sleep/fatigue); n.a., not applicable; ESS, Epworth Sleepiness Scale.

Data of categorical values are presented as n, %. Data of continuous variables are presented as mean ± standard deviation, median (endpoint scores at baseline and randomization) or mean (95% CI), median (change of endpoint scores within a group or the difference of changes between groups).

^a^ Within‐group comparison.

^b^ Between‐group comparison. For all p‐values, significance level was set at p ≤ 0.05.

*P‐value corrected for multiple testing (multiplied by 2). Endpoints during the double‐blind phase were analyzed separately for the nabilone and placebo groups using a Wilcoxon matched‐pairs test for within‐group comparison (correction for multiple comparisons with a factor of 2) and a Mann–Whitney U test for between‐group comparisons. **Effect size according to Cohen's D with Hedges' g correction. ***Effect size according to Cohen's D with Hedges’ correction/ Common Language Effect Size (CLES). **** χ^2^ test.

For MDS‐UPDRS‐1.7, −1.8, and ESS: see legend of Table 1.

NMSS Domain 2 questions:

Q 3. Does the patient doze off or fall asleep unintentionally during daytime activities? (For example, during conversation, during mealtimes, or while watching television or reading).

Q 4. Does fatigue (tiredness) or lack of energy (not slowness) limit the patient's daytime activities?

Q 5. Does the patient have difficulties falling or staying asleep?

Q 6. Does the patient experience an urge to move the legs or restlessness in legs that improves with movement when he/she is sitting or lying down inactive?

During DB drug withdrawal, MDS‐UPDRS‐1.7 and NMSS‐D2 scores deteriorated less in the nabilone group compared to placebo resulting in significant between‐group differences (*P* < 0.001 and *P* = 0.011; effect sizes: 1.65 and 1.00 (Cohen's D)). Importantly, only patients switched to placebo deteriorated significantly in MDS‐UPDRS‐1.7 and NMSS‐D2 during DB withdrawal (*P* = 0.004), while scores of patients on nabilone remained stable (*P* = 1.000 and *P* = 0.800, Table 2). During the DB phase, five patients (29.4%) on nabilone compared to 12 patients (85.7%) on placebo worsened by at least one point in the MDS‐UPDRS‐1.7 (*P* = 0.002), and one patient (5.9%) on nabilone compared to 11 patients (78.6%) on placebo by at least two points (*P* < 0.001, Table 2). Noteworthy, of the 24 patients with no relevant sleep problem (i.e., MDS‐UPDRS‐1.7 ≤ 1) at randomization, only two patients in the nabilone group (16.7%) compared to 11 patients in the placebo group (91.7%, *P* < 0.001) deteriorated during the DB withdrawal and suffered from clinically‐relevant sleep problems with a score of ≥2 points on MDS‐UPDRS‐1.7 at the termination visit (Table 2). The effects of nabilone on the NMSS‐D2 were mainly reflected by improvements of Q5 of the NMSS (i.e., difficulty falling or staying asleep), while neither Q3 (i.e., daytime sleepiness), Q4 (i.e., fatigue), nor Q6 (i.e., restless legs) deteriorated without nabilone. This is supported by results of the analyses of MDS‐UPDRS‐1.8 and the ESS (no significant within‐ or between‐group differences, Table 2). A multivariate regression analysis (including group, difference in Hoehn and Yahr stage, difference of MDS‐UPDRS‐1.4, and difference of MDS‐UPDRS‐3) revealed group distribution to be the only significantly and independently associated factor with both the difference of the MDS‐UPDRS‐1.7 and NMSS‐D2 between randomization and termination.

## Discussion

Sleep problems in PD are multifactorial including primary dysfunction in the regulation of the sleep–wake cycle related to neurodegeneration, as well as secondary effects of parkinsonian motor and NMS on sleep onset and maintenance, effects of PD medications on sleep and wakefulness, and comorbid conditions such as restless legs syndrome/ periodic limb movements of sleep or sleep‐disordered breathing.^2^, ^10^, ^11^ The diagnosis of insomnia is based on patients’ reporting which often comprises difficulties falling asleep or maintaining sleep, early morning awakening, non‐restorative sleep and consequently impairment of daily activities.^2^ Thus, treatment of PD‐related sleep problems is usually complex including improvement of nocturnal motor symptoms, reduction of daytime sleepiness, targeting other common NMS such as nocturia, depression, sleep hygiene, as well as the addition of sleep promoting drugs.^2^

The potential therapeutic effect of cannabinoids on NMS in PD is a prominent topic raised by patients^12^ and has recently gained increasing interest in the scientific community, although the mechanism of action are still not fully understood and studies on the use of cannabinoids for sleep problems in PD patients are scarce.^13^, ^14^

Preclinical and clinical studies on the efficacy of cannabinoids on sleep yield conflicting results and effects vary according to dose and duration. However, there is evidence for a decrease in sleep latency with the short‐term use of THC in patients with insomnia. Moreover, an improvement of total sleep time, quality, and nightmares in patients with posttraumatic stress disorder using nabilone was observed.^15^, ^16^ Possible mechanisms are modulation of the monoaminergic, GABA‐ergic, glutamatergic, and opioid signaling via the ascending reticular activating system.^15^, ^17^, ^18^, ^19^, ^20^, ^21^

There are several limitations to consider. The post‐hoc selection using MDS‐UPDRS‐1.7 may have biased the results. Moreover, the assessment of OL responders in an EERW trial may reduce generalizability or lead to an overestimation of the study drug's efficacy. Still, most of our PD patients were OL responders in the main trial and treatment of responders only reflects clinical practice in line with personalized medicine. An inherent limitation of subgroup analyses is often a small sample size. In our study, however, most patients of the original trial population^3^ were considered for the subgroup analysis (31/38 patients (i.e., 81.6%) randomized in the main trial). As post‐hoc evaluations are exploratory in nature, a power calculation was not performed. The results of post‐hoc analysis are observatory and as such cannot conclusively determine the (statistical) effects of nabilone on sleep problems in the overall PD population. Also, our study lacks video‐PSG outcome measures to assess sleep objectively. The outcome measures used are patient‐reported and therefore represent a patient‐centered approach. Nevertheless, it is not possible to disentangle whether sleep problems result from pure insomnia, nightly motor discomfort, or other NMS such as pain, nocturia, or neuropsychiatric disturbances. However, as assessed with multiple regression analysis, the effect of nabilone seems independent of anxiety (MDS‐UPDSR‐1.4), motor symptoms (MDS‐UPDRS‐3), and disease stage (Hoehn and Yahr). Our trial did not include further scales or questionnaires for the assessment of sleep problems, because we did not expect this effect of nabilone on sleep a‐priori when planning the NMS‐Nab trial. The outcome measures used in this post‐hoc analysis are known to have moderate to strong correlations with other rating scales commonly used to detect sleep problems in PD, such as the Pittsburgh Sleep Quality Index (PSQI) or Parkinson's Disease Sleep Scale (PDSS).^22^, ^23^

Finally, the negative expectation of participants to receive placebo during the withdrawal phase may lead to an underestimation of the effects of tested drug (i.e., “lessebo effect”^24^). This may be the reason for the non‐significant deterioration of various outcome variables (e.g., single NMS of the MDS‐UPDRS‐1) in the nabilone group in phase 2, as shown in the main analysis of this trial^3^ and this post‐hoc analysis (Table 2).

With the study's EERW design, long‐term exposure to the study drug can be limited by early discontinuation in case of deterioration thus reducing harm through a possibly ineffective treatment. Moreover, total exposure to placebo is reduced compared to standard randomized controlled trials, individualized dosing regimens can be implemented so that the assessment of dose–response relations is possible, and lastly reduction of sample size without jeopardizing data quality must be named as an advantage of the EERW trial design. All these provide an enhanced benefit–risk relationship for participants.^6^, ^25^, ^26^

Despite the limitations, we found positive effect of nabilone on clinically‐relevant sleep problems in PD.

## Author Roles

1. Research project: A. Conception, B. Organization, C. Execution;
2. Statistical Analysis: A. Design, B. Execution, C. Review and Critique;
3. Manuscript Preparation: A. Writing of the first draft, B. Review and Critique;

MP: 1A, 1B, 1C, 2A, 2B, 3A, 3B

KS: 1A, 1B, 1C, 2A, 2B, 3A, 3B

FK: 1C, 2B, 2C, 3B

AD: 1A, 1C, 3A, 3B

HGK: 1C, 3B

SS: 1C, 3B

BH: 1C, 3B

PE: 1C, 3B

MW: 1C, 3B

WP: 1A, 1B, 3B

## Disclosures

**Ethical Compliance Statement:** The NMS‐Nab study was approved by the ethics committee of the Medical University of Innsbruck and the Austrian national regulatory authorities. All patients gave written informed consent prior to participate in the study. On behalf of all co‐authors, the first and corresponding authors confirm that all authors have read the Journal's position on issues involved in ethical publication and affirm that this work is consistent with those guidelines.

**Funding Sources and Conflicts of Interest:** AOP Orphan Pharmaceuticals AG manufactured the study drug and placebo and provided compensation of in‐person study visits and the independent monitoring conducted by the Clinical Trial Centre of the Medical University of Innsbruck. AOP Orphan Pharmaceuticals AG had no further role in study design, in the collection, analysis and interpretation of data, in the writing of the report, and in the decision to submit the paper for publication. KS reports personal fees from AOP Orphan Pharmaceuticals AG outside the submitted work. MP, AD, WP, FK, HGK, PE, MW, and SS have no relevant financial or non‐financial interests to report in relation to the manuscript.

**Financial Disclosures for the Previous 12 Months:** MP, PE, FK, HGK, SS and MW declare that there are no additional disclosures to report. A.D. reports honoraria from Novo Nordisk, Roche, Biogen and Abbvie.

BH reports honoraria from Novartis AG outside the submitted work.

KS reports honoraria from the International Parkinson and Movement Disorders Society, grants from the FWF Austrian Science Fund, the Michael J. Fox Foundation, and the International Parkinson and Movement Disorder Society, as well as personal fees from Teva, UCB, Lundbeck, AOP Orphan Pharmaceuticals AG, Abbvie, Roche, and Grünenthal outside the submitted work.

WP reports personal consultancy and lecture fees from: Alterity, AbbVie, Affiris, BIAL, Biogen, Britannia, Lilly, Lundbeck, Neuroderm, Neurocrine, Denali Pharmaceuticals, Novartis, Orion Pharma,

Roche, Takeda, Teva, UCB and Zambo Royalties: Thieme, Wiley Blackwell, Oxford University Press and Cambridge University Press Grant Support: MJFF; EU FP7, & Horizon 2020.

## Availability of Data and Material

The study protocol with the statistical analysis plan, informed consent form, and study data, including deidentified participant data will be made available to other researcher upon formal request and after approval of the proposal and receipt of a signed material transfer agreement. Only deidentified individual data that underlie the results reported in this manuscript will be made available (text, tables, figures, supplemental material). Proposals should be directed to the first authors. Data will be available beginning three months and ending five years following article publication solely for the purpose of achieving aims in the approved proposal. Data will only be shared via individual secured network connections.

## Supporting information


**Supplementary Table S1.** Changes of MDS‐UPDRS‐1.7 (“sleep problems”) and NMSS Domain 2 (“sleep/fatigue”) in PD patients with clinically‐relevant sleep problems during the trial
