# Supplementary material for: Effects of Nabilone on Sleep Outcomes in Patients with Parkinson's Disease: A Post‐hoc Analysis of NMS‐Nab Study
Source: Mov Disord Clin Pract. 2022 May 31;9(6):751–8. doi: 10.1002/mdc3.13471 (PMC9346252; doi:10.1002/mdc3.13471)
Supplement: Supplementary file 1 — Supplementary Table S1. Changes of MDS‐UPDRS‐1.7 (“sleep problems”) and NMSS Domain 2 (“sleep/fatigue”) in PD patients with clinically‐relevant sleep problems during the trial [file MDC3-9-751-s001.docx]

**Supplementary Table 1: Changes of MDS-UPDRS-1.7 ("sleep problems”) and NMSS Domain 2 ("sleep/fatigue”) in PD patients with clinically-relevant sleep problems during the trial**

|  | **MDS-UPDRS-1.7** | | **NMSS Domain 2** | |
| --- | --- | --- | --- | --- |
| **Open-label Phase** | | | | |
| Baseline (BL) | 2.87 ±0.85, 3.00 | | 15.32 ±7.72, 14.00 | |
| Randomization (R) | 0.90 ±1.01, 1.00 | | 9.55 ±7.92, 8.00 | |
| Change from BL to R | -1.97 (-2.30; -1.63) | | -5.77 (-8.24; -3.31) | |
| p-value | <0.001 | | <0.001 | |
| **Double-blind Phase** | | | | |
|  | **Placebo (n=14)** | **Nabilone (n=17)** | **Placebo (n=14)** | **Nabilone (n=17)** |
| Randomization (R) | 0.79 ±1.12, 1.00 | 1.00 ±0.94, 1.00 | 7.57 ±7.06, 6.00 | 11.18 ±8.43, 8.00 |
| Termination visit (T) | 2.79 ±1.05, 3.00 | 1.12 ±1.11, 1.00 | 17.14 ±11.72, 14.00 | 12.18 ±8.67, 10.00 |
| Within-group change from R to T | 2.00 (1.32; 2.68) | 0.12 (-0.45; 0.69) | 9.57 (3.24; 15.90) | 1.00 (-2.08; 4.08) |
| p-value **^a,*^** | 0.004 | 1.000 | 0.004 | 0.800 |
| Between-group difference | 1.88 (1.04; 2.73) | | 8.57 (2.24; 14.91) | |
| p-value **^b^** | <0.001 | | 0.011 | |
| Effect size **^a,**^** | 1.70 | 0.11 | 0.87 | 0.17 |
| Effect size **^b,***^** | 1.65/ 0.88 | | 1.00/ 0.76 | |

Abbreviations: BL, baseline; R, randomization; T, termination visit; ES, MDS-UPDRS, Movement Disorder Society- Unified Parkinson´s Disease Rating Scale; NMSS, Non-Motor Symptoms Scale; 1.7, MDS-UPDRS-1 item 1.7 (Sleep problems); D2, NMSS Domain 2 (sleep/fatigue). Data of continuous variables are presented as mean ± standard deviation, median (endpoint scores at baseline and randomization) or mean (95% CI), median (change of endpoint scores within a group or the difference of changes between groups). ^a^Within-group comparison. ^b^ Between-group comparison. For all p-values, significance level was set at p≤0.05. ^*^ p-value corrected for multiple testing (multiplied by 2). Endpoints during the double-blind phase were analyzed separately for the nabilone and placebo groups using a Wilcoxon matched‐pairs test for within‐group comparison (correction for multiple comparisons with a factor of 2) and a Mann–Whitney U test for between‐group comparisons. ^**^Effect size according to Cohen´s D with Hedges’ g correction. ^***^Effect size according to Cohen´s D with Hedges’ correction / Common Language Effect Size (CLES). For MDS-UPDRS-1.7: see legend of Table 1.
